# Different Dynamics in 6aJL2 Proteins Associated with AL Amyloidosis, a Conformational Disease

**DOI:** 10.3390/ijms20174078

**Published:** 2019-08-21

**Authors:** Roberto Maya-Martinez, Leidys French-Pacheco, Gilberto Valdés-García, Nina Pastor, Carlos Amero

**Affiliations:** 1 Laboratorio de Bioquímica y Resonancia Magnética Nuclear, Centro de Investigaciones Químicas, Instituto de Investigación en Ciencias Básicas y Aplicadas, Universidad Autónoma del Estado de Morelos, Cuernavaca 62209, Mexico; 2 Centro de Investigación en Dinámica Celular, Instituto de Investigación en Ciencias Básicas y Aplicadas, Universidad Autónoma del Estado de Morelos, Cuernavaca 62209, Mexico

**Keywords:** amyloidosis, nuclear magnetic resonance, immunoglobulin light-chain, protein dynamics

## Abstract

Light-chain amyloidosis (AL) is the most common systemic amyloidosis and is caused by the deposition of mainly insoluble immunoglobulin light chain amyloid fibrils in multiple organs, causing organ failure and eventually death. The germ-line λ6a has been implicated in AL, where a single point mutant at amino acid 24 (6aJL2-R24G) has been observed in around 25% of patient samples. Structural analysis has shown only subtle differences between both proteins; nevertheless, 6aJL2-R24G is more prone to form amyloid fibrils. To improve our understanding of the role of protein flexibility in amyloid fibril formation, we have used a combination of solution nuclear magnetic resonance spectroscopy and molecular dynamics simulations to complement the structural insight with dynamic knowledge. Fast timescale dynamics (ps–ns) were equivalent for both proteins, but suggested exchange events for some residues. Even though most of the intermediate dynamics (μs–ms) occurred at a similar region for both proteins, the specific characteristics are very different. A minor population detected in the dispersion experiments could be associated with the formation of an off-pathway intermediate that protects from fiber formation more efficiently in the germ-line protein. Moreover, we found that the hydrogen bond patterns for both proteins are similar, but the lifetime for the mutant is significantly reduced; as a consequence, there is a decrease in the stability of the tertiary structure that extends throughout the protein and leads to an increase in the propensity to form amyloid fibers.

## 1. Introduction

Light-chain amyloidosis (AL) is a deadly disease, caused by the deposition of immunoglobulin light chain (LC) amyloid fibrils in organs, resulting in organ failure and eventually death [1]. It is the most common systemic amyloidosis (~70%) and is triggered by a proliferative monoclonal population of plasma cells that overexpress LC proteins [1,2]. Once these immunoglobin LC proteins are in the bloodstream, they misfold and aggregate as amyloid fibrils in tissues and organs, affecting, most frequently, kidneys (52%), heart (25%), liver (8%), and peripheral nervous system (8%). AL has been described as very cytotoxic, generating increased oxidative stress that results in apoptosis and cell death [3]. The average age at diagnosis is around 65 years and once the disease becomes symptomatic the median survival is of 4 years [4]. 

Two identical LC proteins combine with two identical heavy chain proteins to form an antibody. While there are five classes of heavy chain proteins, there are only two classes of LC proteins: kappa and lambda [5]. Each class of LC proteins has several dozen variants that are further classified into different germ-lines. In addition to the diversity in class and variants, the LC contains variable gene segments, the section that codes for the region that recognizes antigens, which are combined with a joining and a constant segment and then undergo a somatic hypermutation process [6,7]. As a result, there is an enormous heterogeneity of LC proteins. 

**Figure 1:**
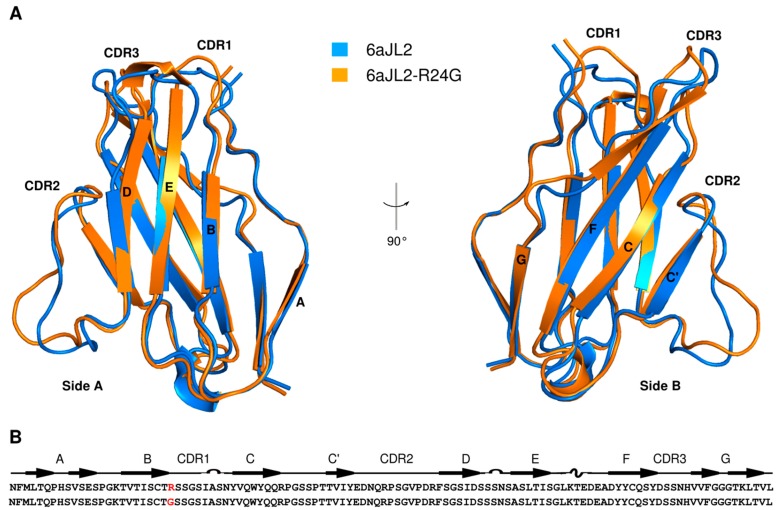
(**A**) Ribbon diagram of the variable domain of the light chain lambda 6a germ-line protein in blue (6aJL2; PDB entry 2MMX) and its single point mutant at amino acid 24 in orange (6aJL2-R24G; PDB entry 2MKW) superposed on the backbone heavy atoms. Side A (strands: D,E,B, and A) and side B (strands: G,F,C, and C’) and the three complementary determining regions (CDR1, CDR2, and CDR3) are labeled. Images were rendered with PyMOL. (**B**) Secondary structure elements and sequence of the constructs. The mutation site is shown in red.

In general, healthy individuals have a kappa to lambda ratio of two to one while AL patients have a kappa to lambda ratio of one to three [3], with a majority of the lambda light chains coming from only a few possible germ lines, such as 3r and/or 6a [8]. Particularly, a mutation of Arg to Gly in position 24 for germ-line 6a has been found in 25% of AL patient samples. The canonical structure of the LC variable domains consists of a β-sandwich fold composed of eight β-strands (named A, B, C, C’, D, E, F and G) tightly packed against one another and linked by a disulfide bridge (Figure 1) [9]. Different studies have shown that one of the effects of the R24G mutation is an increase in the propensity of the protein to aggregate as amyloid fibrils in vitro [10,11,12], mainly manifested as a reduction in lag time for fibril formation. The first attempts to understand this difference were focused in structural analysis. However, a comparison of the 6aJL2 and 6aJL2-R24G structures deposited in the Protein Data Bank (PDB) did not show significant differences with a root-mean-squared deviation (RMSD) of 0.9 Å (Figure 1) (PDB entries 2MMX and 2W0K for 6aJL2; 2MKW, 5JPJ, and 5C9K for 6aJL2-R24G), [13,14,15]. The reduction of the lag time has been ascribed to the instability provoked by the loss of the cation-pi interaction between Phe2 and Arg24 [11,16,17] and by local structural rearrangements resulting from this loss [18]. 

As a structural approach cannot fully explain the differences in stability and fiber formation kinetics for the light chains lambda 6a germ-line protein (6aJL2) and its single point mutant at amino acid 24 (6aJL2-R24G), we decided to explore the dynamic signatures of both proteins, using high-resolution Nuclear Magnetic Resonance (NMR) techniques complemented with molecular dynamics simulations (MD). Taking into account that the motions studied here occur on different timescales, and on specific parts of the proteins, this study would allow us to understand the contribution of the dynamics of these AL model proteins in amyloid fibril formation.

## 2. Results

Previous studies have established that LC proteins are able to adopt a monomeric or dimeric conformation. To assess the main oligomeric state of the λ6a proteins, we measured the diffusion coefficients by Dynamic Light Scattering (DLS). The theoretical translational diffusion coefficient predicted by hydrodynamic calculations from the coordinates of 6aJL2 (PDB entry 2MMX) and 6aJL2-R24G (PDB entry 2MKW) with hydroPRO [19] software were 1.263 × 10^−7^ cm^2^/s and 1.261 × 10^−7^ cm^2^/s, respectively. DLS measurements of the proteins showed an apparent radius of ~2.1 nm and ~2.2 nm corresponding to a diffusion coefficient of ~1.17 × 10^−7^ cm^2^/s and ~1.11 × 10^−7^ for 6aJL2 and 6aJL2-R24G respectively; this shows a good agreement with the theoretical values (Figure S1). This result suggests that the two proteins are mostly in a monomeric state under the tested conditions. 

Although the three-dimensional structure of both proteins is very similar (a backbone RMSD of 0.9 Å), their amyloid propensities are very different. In order to improve our understanding of the role of protein flexibility in amyloid fibril formation, it is necessary to complement the structural insight with dynamic knowledge. We have, therefore, characterized the motion at different timescales. 

### 2.1. Fast Timescale Backbone Dynamics

We measured the effect of bond motions on the longitudinal relaxation rate (R_1_), the transverse relaxation rate (R_2_), and the heteronuclear cross-relaxation rate (HetNOE). The mean values for R_1_, R_2_, and HetNOE obtained at 700 MHz and 25 °C are presented in Table 1. The high HetNOE and the relatively uniform R_2_ and R_1_ for both the germ-line and the mutant are indicative of a highly ordered structure at this timescale (Figure S2). A rotational correlation time (t_c_) of 10.7 and 11 were estimated based on the R_2_/R_1_ ratio for each construct. Accordingly, these values were consistent with the presence of monomeric proteins. 

**Figure 2:**
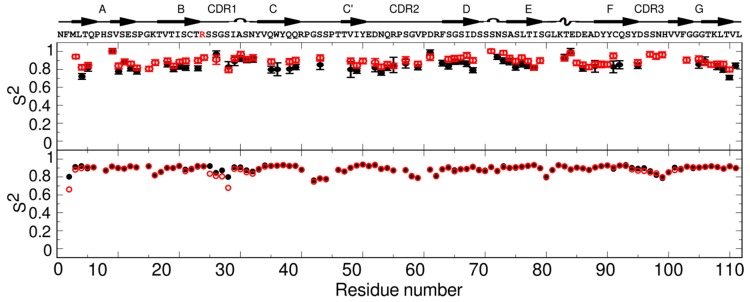
Fast timescale dynamics of 6aJL2 (black) and 6aJL2-R24G (red). Top panel: Model Free experimental order parameters S^2^ reflecting ps–ns motions. Bottom panel: Calculated order parameters from molecular dynamic (MD) simulations. The protein sequence and secondary structure elements are indicated above the top panel. The mutation site is shown in red.

Residue specific rates for both proteins showed that there is not a significant difference (larger than twice the error) for most amino acids, indicating very similar backbone dynamics (Figure S2). To separate the internal motion from the global tumbling, we used the model-free analysis formalism. The data were fit by non-linear least-squares to obtain parameters that describe the motion. Model-free results for 6aJL2 were obtained for 58 backbone amides (out of 91 possible, excluding prolines and not assigned residues). Of these, the relaxation rates for 19 amides were best fit by model 1 (S^2^) and 39 by model 3 (S^2^ and R_ex_). No residue was fit using model 2 (S^2^ and t_e_), model 4 (S^2^, t_e_ and R_ex_), or model 5 (S^2^_f_, S^2^_s_, t_e_ and t_m_). The backbone of 6aJL2 shows restricted mobility on the ns–ps timescale, with an average S^2^ value of 0.84 ± 0.06 (Figure 2).

In the case of 6aJL2-R24G, dynamics information was obtained for 69 backbone amides (out of 95 possible). Relaxation data for 15 amides were best fit by model 1 and 54 by model 3, and none by models 2, 4, and 5. The overall ps–ns dynamics are similar to those of the 6aJL2 protein, with an average S^2^ of 0.88 ± 0.05 (Figure 2). 

These results show that both proteins experience similar internal backbone motions, implying no large changes in the ps–ns dynamics due to the mutation. Interestingly, inspection of the models needed to describe the data reveals several residues that require an extra R_ex_ parameter; this term is an indication that motions at a slower timescale were required to fit the relaxation data (Figure S3). Even though R_ex_ is not usually used to provide precise values for the exchange time, it strongly suggests the presence of internal motions on the μs–ms timescale. 

### 2.2. Molecular Dynamics

In order to get a deeper atomic description and take into account side chain motions, we compared the experimental data with previously reported MD simulations [18]. As previously shown, the correlation between calculated chemical shifts for alpha carbon (CA) and beta carbon (CB) with experimental data was high. 

Per residue differences in the MD calculated generalized order parameter S^2^ for the backbone N−H bonds between 6aJL2, and the mutant showed a global loosening of the protein, where the CDR1 (residues 25 to 34) and N-terminal residues were the most affected. Comparisons between MD and NMR data are shown in Figure 2. The average MD S^2^ obtained for the constructs (0.89) are similar to those obtained by NMR (0.84 and 0.88), reflecting restricted motions in the ps–ns timescale. The main discrepancy is observed around the mutation site, where MD-derived S^2^ are lower than those obtained from NMR for the two proteins. As reported before, the side chain dynamics show changes in the hydrophobic core [18].

From the MD simulations, we also extracted the residues that were involved in intramolecular hydrogen bonds and residues that were buried over the trajectory time. For both proteins, the NH groups in the strands C’, C, F, and G at side B were more protected because of its concave shape and the presence of an aromatic patch.

### 2.3. Medium Timescale Backbone Dynamics

The μs–ms timescale dynamics were probed by Carr-Purcell-Meiboom-Gill (^15^N-CPMG) relaxation dispersion NMR experiments. The data for individual residues were fit to the Carver-Richards slow two-site exchange model, only considering residues with R_ex_ > 3 s^−1^ as residues with significant dispersion (Figures S4 and S5). 

**Figure 3:**
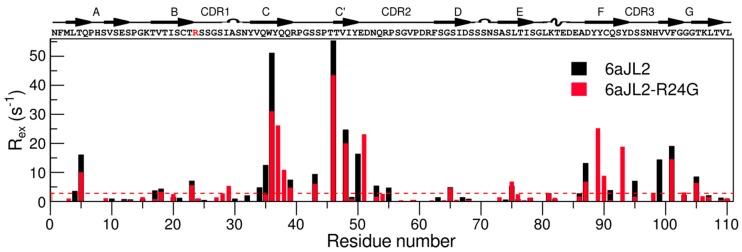
Medium timescale dynamics of 6aJL2 (black) and 6aJL2-R24G (red). Plot of R_ex_ as a function of residue number reflecting μs–ms motions. The dotted line represents R_ex_ = 3 s^−1^. The protein sequence and secondary structure elements are indicated above the top panel. The mutation site is shown in red.

Analysis of the data for 6aJL2 revealed 23 of the 67 backbone amides tested go through chemical exchange. Most of the residues with exchange are located in the side B of the protein (strands G, F, C, C’) and the CDR2, whereas most of the residues in side A (strand D, E, B, A) did not present exchange (Figure 3). 

For the mutant 6aJL2-R24G, only 22 residues out of the 70 possible exhibit relaxation dispersion. Notably, most of these residues are located in similar regions as in the germ-line protein (Figure 3). Moreover, some of the most dynamic residues are the same in both proteins (T5, W36, Y37, T46, V48, and V101), suggesting that each of the proteins undergoes conformational exchange between the ground state and a common excited state. Nevertheless, there is a clear change in the dynamics parameter for both proteins; overall, 6aJL2 presents a larger R_ex_ as defined by the difference between R_2,eff_ values at the lowest and highest νCPMG values (Figure 3). 

More quantitative dynamics parameters, including exchange rate (k_ex_) and population of the minor conformer (p_B_), were extracted for the different constructs by fitting the ^15^N-CPMG relaxation dispersion trajectories to a two-site exchange model. Our results showed similar values for most of the residues in each protein, suggesting that these amides exchange simultaneously rather than experiencing completely individual uncorrelated motions. Therefore, we simultaneously fit all of them to a single exchange rate and minor conformer fraction (Table 2). Representative calculated trajectories corresponding to this fitting procedure are shown in Figure S5.

These data indicate that 6aJL2 has an exchange rate in the order of 1000 s^−1^ with a minor conformer population of 15%, whereas for the mutant protein the exchange rate increases with a small decrease in the population (Table 2).

### 2.4. Hydrogen Deuterium Exchange

**Figure 4:**
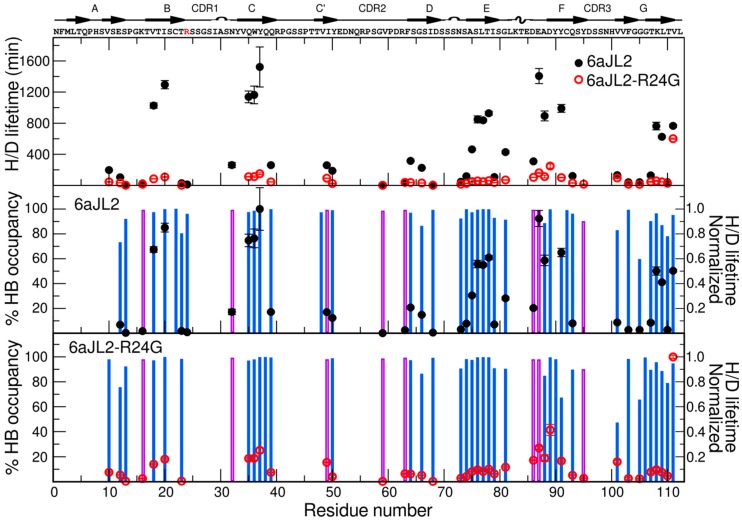
Hydrogen deuterium mean exchange lifetime values compared to hydrogen bond occupancies. Top panel: Experimental Hydrogen deuterium (H/D) exchange lifetimes reflecting the slow motions of 6aJL2 (black) and 6aJL2-R24G (red). Middle panel: Percentage of backbone N−H hydrogen bond occupancy calculated from MD simulations (blue bars). Black circles represent the normalized H/D lifetimes for 6aJL2. Purple bars represent the percentage of buried residue from 0 Å exposed (100% buried) to 10 Å exposed (0% buried). Bottom panel: Percentage of backbone N−H hydrogen bond occupancy calculated from MD simulations (blue bars). Red circles represent the normalized H/D lifetimes for 6aJL2-R24G. Purple bars represent the percentage of buried residue from 0 Å exposed (100% buried) to 10 Å exposed (0% buried). The protein sequence and secondary structure elements are indicated above the top panel. The mutation site is shown in red.

To obtain dynamic information in longer timescales, we performed hydrogen-deuterium exchange experiments, in which the intensities of correlations in a series of 2D ^1^H-^15^N HSQC spectra were monitored as a function of time after addition of D_2_O to lyophilized protein. The exchange rate was calculated from the change in intensities of the successive spectra. For the 85 possible amides for 6aJL2, 43 were lost before our first data point, 11 showed exchange times values below 100 s, 16 showed times between 100 and 600 s and 14 residues had times above 600 s. On the other hand, for the 6aJL2-R24G, from 85 possible peaks, 44 residues exchanged before our first data point, 31 had exchange times values below 100 s, 8 showed times between 100 and 600 s, and only one had a times above 600 s (Figure 4). 

Even though most of the exchanging residues for both proteins are located in the same regions (Figure 4), the H/D exchange times highlight some important and significant differences. Residues V18, I20, Q35-Y37, L76-I78, A87, D88, C91, L108, T109 and L111, have higher exchange time than 600 s for 6aJL2, whereas the same residues in 6aJL2-R24G exchange at faster times than 200 s, except for L111. These residues are localized on strands B, E, C, and F. Similar differences are observed in residues V10, E12, N32, Q39, I49, Y50, S64, S66, A74, S75, S79, L81, E86, S93, V101, and K107, which exchange with medium times (between 100 and 600 s) for 6aJL2, whereas the residues in 6aJL2-R24G are less protected and exchange in faster times than 100 s. These residues are located mainly on the CDR1, strands A, B, D, F, C, and C’ (Figure 4).

The percentage of hydrogen bond occupancies during the complete simulation presented a small change between the two proteins. Interestingly, all the amino acids with lower measured H/D exchange rates correspond to those that form H-bonds or are buried during the simulation (Figure 4). 

**Figure 5:**
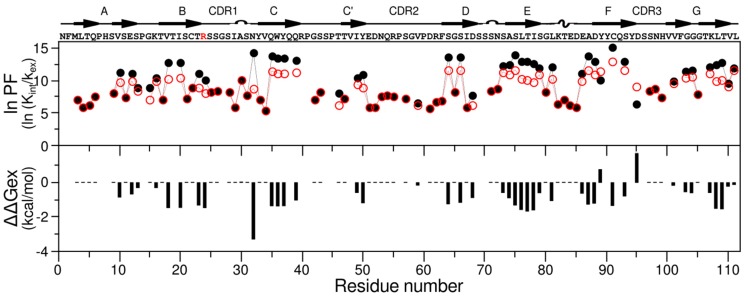
Stabilities as reported for the H/D experiments of 6aJL2 (black) and 6aJL2-R24G (red). Top panel: Plot of protection factors as a function of residue number. Bottom panel: Difference in stabilities due to the mutation R24G. Protein sequence and secondary structure are indicated above the top panel. The protein sequence and secondary structure elements are indicated above the top panel. The mutation site is shown in red.

To evaluate the relative stabilities of the domains, per-residue, the exchange rates k_H/D_, were subsequently converted into the protection factor and the ΔΔG values via the relation ΔΔG = –RT ln(P_6aJL2-R24G_/P_6aJL2_). As can be seen in Figure 5, the protection factors were uniformly higher for 6aJL2, and as a consequence, the 6aJL2-R24G construct became less stable, as seen in the ΔΔG values. 

## 3. Discussion

The germ-line λ6a has been implicated in light chain amyloidosis disease, where a mutation at position 24 of one arginine to a glycine has been observed in around 25% of patient samples [17]. Structural analysis has shown only subtle structural differences between both proteins; however, the amyloid fibril propensities are completely different. Stability experiments with temperature, chaotropic agents, and pH have shown that the 6aJL2-R24G protein is less stable than the germ-line [17,20]. The difference in the stability has been associated with the loss of an interaction between residue F2 and R24. However, the structural changes are not enough to explain the amyloidogenic difference [13]. Recently, it has been shown that dynamics information is important to have a better understanding of the protein fibril aggregation propensities [18,21,22]. Here, we complement the structural work with experimental and simulation dynamic approaches. 

Light chain proteins are often found forming dimers, and while this particular lambda light chain has been reported as a monomer, we have investigated the oligomeric state of 6aJL2 and 6aJL2-R24G by DLS [23]. The predicted translational diffusion coefficient from the structures (~1.26 × 10^−7^ cm^2^/s) agrees well with DLS measurements (~1.17 × 10^−7^ cm^2^/s). From these analyses, we conclude that under these conditions the 6aJL2 variants are principally monomeric in solution. 

**Figure 6:**
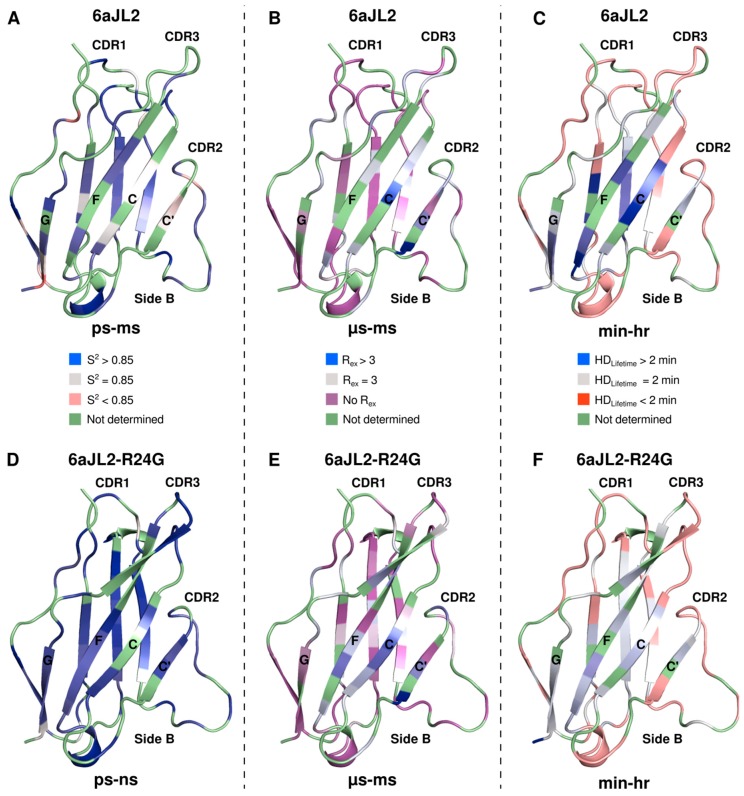
Effect of mutation R24G on dynamics. Fast timescale dynamics for (**A**) 6aJL2 and (**D**) 6aJL2-R24G. Backbone amide ^15^N square of generalized order parameter (S^2^) are mapped with linear color ramps from light gray (S^2^ = 0.85) to red for amides more dynamic than the mean (S^2^ < 0.85) or blue for amides more rigid than the average (S^2^ > 0.85). Residues for which no information was determined are shown in green. Medium timescale dynamics for (**B**) 6aJL2 and (**E**) 6aJL2-R24G. Measured R_ex_ mapped into the cartoon diagram, color coded accordingly to the Rex via a linear gradient ramp from purple (no Rex) to blue (maximum exchange). Residues for which no information was determined are shown in green. Slow dynamics for (**C**) 6aJL2 and (**F**) 6aJL2-R24G. Hydrogen deuterium exchange mean lifetimes mapped to the structure. The magnitude is represented by a linear color ramp from gray (low protected) to blue (maximum protection). Residues colored red exchanged before the first measurement and are the least protected. Residues colored green are those for which the data were not available.

The characterization on the ps to ns timescale motions were performed through ^15^N NMR relaxation experiments. Our data suggest that, overall, both proteins are relatively rigid in this timescale, as expected for proteins that adopt a β-sandwich fold and have similar backbone motions. It is interesting to note that the amino and carboxyl terminal regions are tightly bound to the rest of the structure without too much mobility. R_1_, R_2_, and HetNoe are commonly analyzed by Model Free formalism to separate the global and internal dynamics and provide parameters to describe the motion. The obtained S^2^ values remain similar for both constructs, implying restricted motion for the two proteins (Figure 6). Intriguingly, several residues require an extra parameter R_ex_ to describe their molecular motion, which suggests motion at slower timescales. 

To complement the relaxation data, we compared the experimental results with MD simulations, which report structural and motion information on the same timescale (ps–ns) [24]. The backbone S^2^ parameters obtained by MD simulations showed a similar trend to the ones obtained by NMR, presenting low dynamics for both proteins. Some light differences appear on residue around the mutation, which have been associated with the rearrangement in the contact network that causes a weaker structure for the 6aJL2-R24G protein [18], while the sidechain MD S^2^ shows a mobility increase spreading throughout the protein (CDR1 region, A, C’, and CDR2 region). Interestingly, we observed some sidechain chemical shifts differences for the proteins at residues located in regions far away from the mutation (Figure S6). The ensemble dynamics could be related to a change in the average environment around these residues, in concordance with the MD S^2^ analysis. 

To assess the existence of slow millisecond motion as suggested by the Model Free analysis, we used CPMG dispersion experiments. CPMG experiments are very useful to detect chemical exchange in proteins that present minor different populations, even at low percentage [21]. We found that the same region for both proteins experiences the same type of motion on this timescale. The central part of strands A, B, D, and E, present restricted motions, suggesting that these sheets are in one stable conformation, whereas the strands C’, C, F, and G at side B present exchange for both proteins (Figure 6). Interestingly, there is dynamics in the CDR2 region between the C’ and D strands, which has been described as a protective element for the border of the C’ and D strand and forms part of an anti-aggregation motif [14]. We also found high dynamics in important residues that could act as a clamp in the sandwich borders, as in residues N53, R55, and G65, which help to keep the CDR2 region and D strand joined, and in T5 and T23, which keep together the N-terminus and the B strands. Unsuspectedly, we observed differences in mobility patterns for 6aJL2-R24G at residues very distant to the mutation area, such as W36, Q38, Q39, R40, S44, T46, R62, S79, and D85 (Figure 6). 

The preliminary individual analysis of residue motion shows similar rates, suggesting that all residues experience motion that happens simultaneously rather than in individual, uncorrelated motions. Therefore, we modeled the exchange for all the dynamics of each protein as a single exchange rate and a minor conformer. While both proteins appear to adopt a similar excited state, the kinetics with which they are formed, and the equilibrium populations, differ for each protein. 6aJL2 has an exchange rate of ~1000 s^−1^ with a minor population of ~15%, whereas 6aJL2-R24G has a faster rate (~2000 s^−1^) but a smaller population (~10%). This difference in dynamic signatures could be associated with an alteration of the contact network that affects the formation of an off-pathway intermediate.

In the mutant, the long and charged chain of arginine is lost and, as a consequence, the other side chains can rotate, and some interactions are broken. Therefore, a difference in the mobility for the residues nearby was expected. Surprisingly, the mutation seems to induce and promote a rearrangement of contacts that extends to other parts of the protein. These results are in accordance with the MD simulation that showed that despite the fact that the mutation is located at one edge of the beta sandwich, its effects are felt across the CDR1 to the other side of the molecule [17]. 

In order to extend the dynamics timescale and map hydrogen bond interactions crucial for the architecture and dynamics of these proteins, we performed hydrogen-deuterium exchange experiments. Although the regions that exchange in both proteins are practically the same, their rates are considerably different. Approximately half of the residues of both proteins exchange before our first point. These residues are primary located on the loops that connect all the strands and on strands A B, D, F, C’, and CDR2 region (Figure 6).

When we evaluate the exchange mean lifetimes for each protein, we found that the mean value for 6aJL2 is much higher than that of 6aJL2-R24G. The overall average lifetime of 6aJL2 is ~6 fold higher than that of 6aJL2-R24G. The largest differences are located in small patches on strands B, C, E, and F, at the core of the proteins. Within the face B, we can highlight the following residues in the strand C: residue Q35, W36, and Y37; strand F: residue A87, D88 and C91; strand G: residue L108, T109 and L111; strand A: residue V18, and I20; strand E: residue L76, T77 and I78 (Figure S7). Although the residues involved in the formation of these stable patches are sequentially distant, they are structurally close together (Figure 6).

We calculated, from the MD simulation trajectories, the N−H hydrogen bond occupancy and the accessibility to solvent. Even though the total number of hydrogen bonds was similar between both proteins, their lifetimes were not the same. We found an excellent correlation between residues that are protected from exchange, due to hydrogen bond formation or low accessibility, and the H/D exchange experiments. These results indicate that while both proteins have similar hydrogen bond patterns, and, consequently, the same secondary and tertiary structures, the lifetime of the hydrogen bonds of 6aJL2-R24G is much shorter. The mutation produces a conformational perturbation that is transmitted through the hydrogen bond network throughout the protein. Our NMR data reveal much more substantial perturbations in the hydrogen bonding within the protein than what is evident just from the tridimensional structure.

From the H/D exchange rates, we were able to calculate the relative stability of the domains, per-residue (ΔG), and the difference in stability due to the mutation (ΔΔG). We observed that the consequence of the mutation has a lower stability at the main core of the protein and in some predicted anti-aggregation motifs, resulting in the increase of aggregation propensity of 6aJL2-R24G relative to the germ-line. 

## 4. Materials and Methods 

### 4.1. Protein Expression and Purification 

Recombinant ^15^N-labeled proteins (6aJL2 and 6aJL2-R24G) were transformed in BL21 strain cells and grown in a 2× YT medium at 37 °C and 200 RPM until an optical density (OD 600) of 0.8 was achieved. Then, the cells were transferred to a minimum media supplemented with ^15^N-ammonium chloride. Protein expression was induced by the addition of IPTG 0.8 mM followed by 12 h of incubation. The cell culture was harvested. The pellet was resuspended in a sucrose solution (100 mM Tris, 20% sucrose, 1 mM EDTA, pH 7.4), incubated in ice for 20 min, and centrifuged for 25 min at 4000 rpm. This new pellet was resuspended in 20 mL of cool water and incubated in ice for another 20 min. Finally, the sample was centrifuged at 13,000 rpm for 30 min. The supernatant with the enriched protein fraction was concentrated through centrifugal concentrators and passed through a HiLoad 16/600 Superdex 200 pg gel filtration column previously equilibrated with a phosphate solution (25 mM phosphate, 75 mM sodium chloride, pH 7.4). The purity of 6aJL2 and 6aJL2-R24G proteins was analyzed by SDS-PAGE. Protein concentration was calculated using the molar extinction coefficient provided by the ProtParam server (http://web.expasy.org/protparam/).

### 4.2. Dynamic Light Scattering

DLS measurements were performed on a Malvern Zetasizer Nano ZS, with a scatter angle at *θ* = 173° at 25 °C, using 0.8872 cP for the viscosity, 1.333 for refractive index, and a laser beam of λ = 633 nm. The equilibrium time was of 120 s; five measurements were achieved, each one with 10 runs of 10 s. Data points were used to obtain translational diffusion coefficients through the calculation of decay rates of scattered light (correlation function) [23]. The hydrodynamic radius, R_H_, was obtained from the diffusion coefficient, D, via the Stokes–Einstein equation. The data were analyzed using cumulant analysis in the software package SEDFIT and the figures were done by GUSSI [25,26]. Samples were analyzed at a concentration of 1 mM, same as in NMR experiments, diluted in 25 mM phosphate solution at pH 7.4, with 75 mM NaCl. 

### 4.3. Nuclear Magnetic Resonance 

All NMR data were processed and analyzed with NMRPipe [27] and CARA [28] programs. Data were recorded at 298 K using magnetic field strengths of 16.4, and 18.8 T, corresponding to ^1^H frequencies of 700 and 800 MHz, respectively. NMR spectrometers that were used: (i) Varian 700 MHz spectrometer equipped with a cryogenically cooled triple resonance pulsed field gradient probe, at LANEM Mexico, and (ii) a Bruker 800 MHZ spectrometer equipped with a triple resonance inverse TXI cryoprobe at The Ohio State University (OSU), USA.

Residue-specific backbone amide ^15^N longitudinal (R_1_) and transverse (R_2_) relaxation rate constants were obtained from standard inversion recovery experiments. The recovery delay for both experiments was 2 s. Nine relaxation time points were sampled in random order for each experiment (100, 900, 300, 2000, 500, 1500, 700, 1300, and 200 ms) and (10, 250, 30, 210, 50, 150, 70, 130, and 90 ms) for R_1_ and R_2_, respectively. Relaxation rates were obtained by fitting the intensity changes of each peak to one order exponential. The standard deviation of the baseline spectral noise was taken to be the uncertainty in peak heights, and uncertainties in the fit parameters were obtained from Monte-Carlo simulations with a confidence interval of 0.68 and 500 replicas. 

Steady-state heteronuclear {^1^H}-^15^N nuclear Overhauser enhancement (HetNOE) spectra were acquired in an interleaved fashion with a 2 s recycle delay. The HetNOE values were determined from the ratios of the peak intensities in spectra acquired with and without proton saturation. 

The relaxation data were analyzed using RELAX software with a model-free optimization protocol [29]. Residue were fit to models in which diffusion was assumed to be spherical, oblate, prolate or ellipsoid, and the overall fit of the data to each model was evaluated. The appropriateness of each model was evaluated by use of the Bayesian information criteria analysis and 500 Monte-Carlo simulations, as implemented in the program [30]. For both proteins, 6aJL2 and 6aJL2-R24G, no significant improvement of anisotropic models over the isotropic models was detected for data fitting. Internal dynamics parameters per residue were obtained by fitting the following parameters: t_m_, overall rotational correlation time of the molecule, t_e_, effective correlation time for internal motions, S^2^, square of the generalized order parameter that describes the amplitude of motions, and Rex, a phenomenological exchange term introduced to account for chemical exchange line-broadening (affected by R_2_). Five different models were used: (i) S^2^, t_m_; (ii) S^2^, t_e_, t_m_; (iii) S^2^ and R_ex_, t_m_; (iv) S^2^, t_e_, R_ex_ and t_m_ and (v) S^2^_fast_, S^2^_slow_, t_e_ and t_m_. Residue with significant overlapped signals were excluded. We employed a value of −172 ppm for the ^15^N chemical shift anisotropy and 1.02 Å for the N-H bond distance. 

Carr–Purcell–Meiboom–Gill (CPMG) dispersion experiments were collected with a standard pulse sequence. We measured the decay of transverse in-phase magnetization as a function of the number of refocusing pulses with a constant relaxation delay of 30 ms. Intensities of cross-peaks in dispersion profiles were converted into R_2,eff_ rates according to the equation:

R_2,eff_(v_CPMG_) = −1/T ln[I(v_CPMG_)/I_o_] (1)

where I and I_o_ are peak intensities from spectra recorded in the presence and absence of refocusing, respectively, and T is the delay between successive refocusing pulses. CPMG field strengths were recorded between 33 and 1000 Hz, with a pair of repeats for error analysis.

The data were individually and globally fit to a two-site slow exchange model using the RELAX software [28]. The variation of R_ex_ as a function of the radio-frequency field allows the calculation of the following dynamics parameters: the exchange rate between two conformers (k_ex_), the population of the conformers (p_B_), and the absolute values of chemical shift differences (|Δω|). Only nuclei with dispersion profile sizes R_ex_ = R_2,eff_(0) – R_2,eff_(1000) > 3 s^−1^ were assumed to have significant dynamics and were included in the analysis. Uncertainties were measured from duplicate points. 

Hydrogen Deuterium Exchange (H/D) experiments were started by re-suspending lyophilized proteins in 100% deuterated phosphate buffer pH 7.4 or in 50%/50% H_2_O/D_2_O phosphate buffer to slow down the exchange process. A series of 2D ^15^N-HSQC spectra were acquired continuously every 7 min for a 12 or 24 h total exchange time for the 100% D_2_O sample and every 2 min for the 50%/50% H_2_O/D_2_O. The data obtained for the slow exchange (50%/50%) was normalized to the 100% D_2_O data, obtaining information corresponding to 0.7 min exchange process. 

The ^1^H exchange rates were determined by fitting the change in the intensities decay to a mono exponential function for each peak. Residue that exchanged before the first time point was assigned to the fastest rates measured. 

Site specific changes in thermodynamic stability were obtained from changes in hydrogen/deuterium exchange rates based on

∆*G*_HD_ = −*RT* ln(*P*) = −*RT* ln(*k_e_*/*k_int_*) (2)

where *P* is the protection factors (*P* = *k_e_*/*k_int_*), *k*_e_ is the measured exchange rates, and *k_int_* is the calculated intrinsic exchange rate.

### 4.4. Molecular Dynamics

The simulations were carried out at 298 K with an NPT ensemble (with constant number of particles, N, pressure, P, and temperature, T) as reported previously [18]. Order parameters (S^2^) were calculated using the NMR module within CHARMM 38b1 [31], setting up a tumbling time of 7.5 ns estimated with hydroPRO [19], over simulation intervals of 2.5 ns, and applying a convergence criterion as previously described [32,33]. Hydrogen bonds (2.4 Å cutoff between H and heavy atom, no angle restriction) and solvent exposed areas (1.4 Å probe radius) were calculated for all the simulation time in 100 ps intervals.

## 5. Conclusions

The germ line 6aJL2 and the mutation 6aJL2-R24G have a similar structure, but their dynamic behavior is different, with larger differences at slower timescales. The minor populations detected in the CPMG experiments could be associated with the formation of an off-pathway intermediate that protects from fiber formation more efficiently in 6aJL2 than in 6aJL2-R24G. Even though the hydrogen bond pattern for both proteins is the same, their stability is completely different. These results, together, provide a strong basis for understanding the increase of the aggregation propensity of 6aJL2-R24G relative to the germ-line. First, the mutation results in a shift in the equilibrium between the monomer and the non-productive conformation, but more importantly, decreasing the stability of the monomeric tertiary structure leads to an increasing propensity to form amyloid fibers.

We have observed changes in dynamics in regions far away from the mutation site, showing how a simple disruption of an interaction in one part of the protein could have a large effect at distant regions and at different timescales. The use of complementary information obtained by using experimental data and computational approaches allowed us to obtain more detailed descriptions. 

## Figures and Tables

**Table 1:** Different relaxation rate values for both light chain (LC) proteins.

Mean Value	6aJL2	6aJL2-R24G
R_1_	1.05 ± 0.08 (s^−1^)	1.09 ± 0.06 (s^−1^)
R_2_	17.3 ± 3.3 (s^−1^)	18.9 ± 4.2 (s^−1^)
HetNOE	0.88 ± 0.04	0.88 ± 0.02

Average of relaxation rate constants of the longitudinal relaxation (R_1_), the transverse relaxation (R_2_), and the heteronuclear cross relaxation (HetNOE) measured at 700 MHz.

**Table 2:** Summary of ^15^N CPMG relaxation dispersion data.

	6aJL2	6aJL2-R24G
k_ex_	1175 s^−1^	2008 s^−1^
p_B_	15%	10%
Average Δω	0.8	0.5

## Supplementary Materials

Supplementary materials can be found at https://www.mdpi.com/1422-0067/20/17/4078/s1.

## Abbreviations

AL	Light-chain amyloidosis
NMR	Nuclear Magnetic Resonance
LC	light chain
MD	Molecular dynamics simulations
