# Supplementary material for: Different Dynamics in 6aJL2 Proteins Associated with AL Amyloidosis, a Conformational Disease
Source: Int J Mol Sci. 2019 Aug 21;20(17):4078. doi: 10.3390/ijms20174078 (PMC6747610; doi:10.3390/ijms20174078)
Supplement: Supplementary file 1 [file ijms-20-04078-s001.zip › ijms-496509-SI.pdf]

International Journal of
Molecular Sciences

MDPI

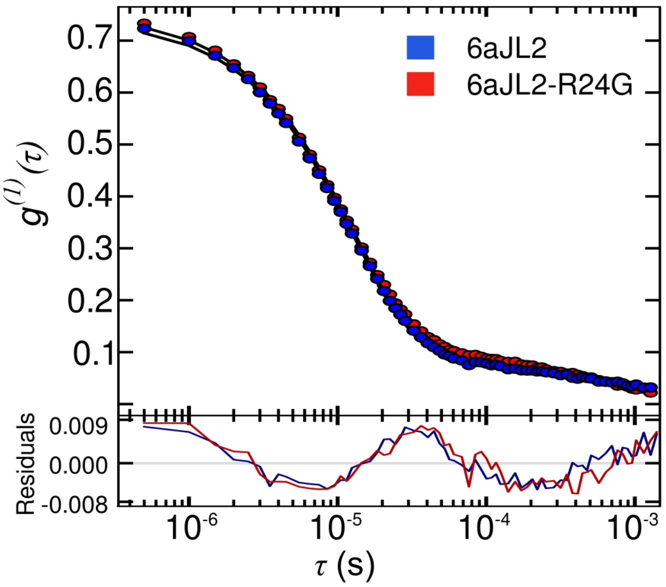

Figure S1. Dynamic light scattering (DLS) analysis. DLS was used to obtain changes in the oligomeric state through measurement of the translational diffusion coefficients. The diffusion coefficient (~1.171 × 10^{-07} cm^2/s) was obtained from the correlation function, in blue (6aJL2) and red (6aJL2-R24G). Two components were used to improve the fit suggesting that a low population with higher radius is also present.

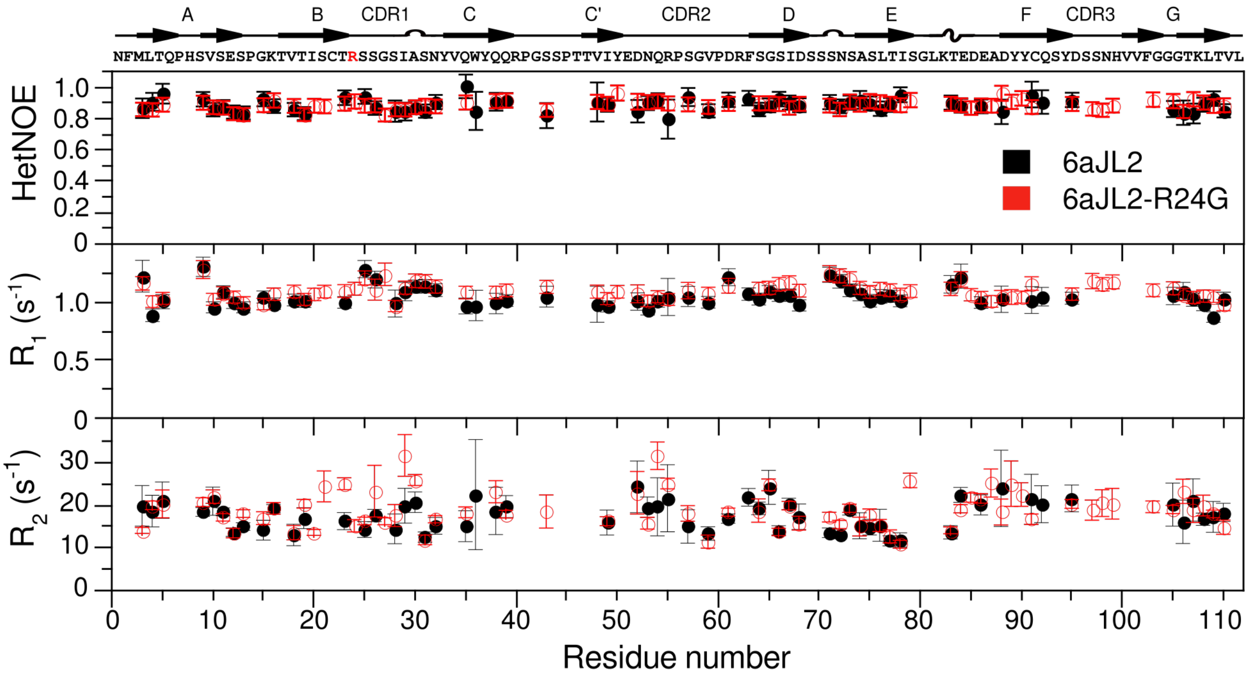

Figure S2. Backbone amide ^{15}N spin relaxation measurements for 6aJL2 (black) and 6aJL2-R24G (red) by residues. Plots of ^{1}H–^{15}N heteronuclear NOE, ^{15}N R_1, and ^{15}N R_2 as a function of residue number. The relaxation measurements were performed at 700 MHz, 25 °C. The secondary structure is indicated above the top panel.

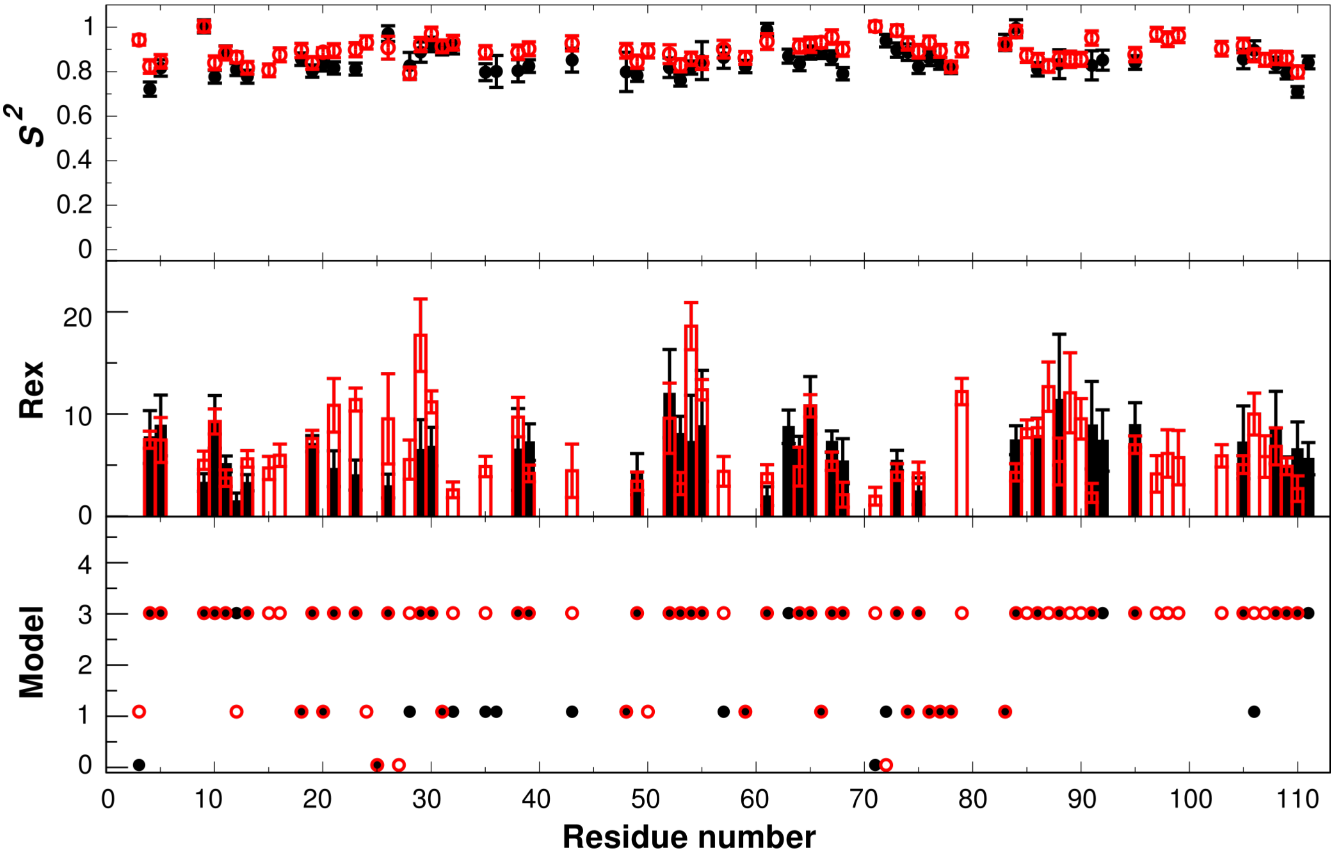

Figure S3. Analysis of fast scale backbone motion of 6aJL2 (black) and 6aJL2-R24G (red) by residues. Order parameter S^2 and Rex parameters were obtained by Model Free analysis doing by fitting ^{15}N spin relaxation (R_1, R_2, and NOE) data at 700 MHz 1H frequency to one of the five possible motion models of RELAX software.

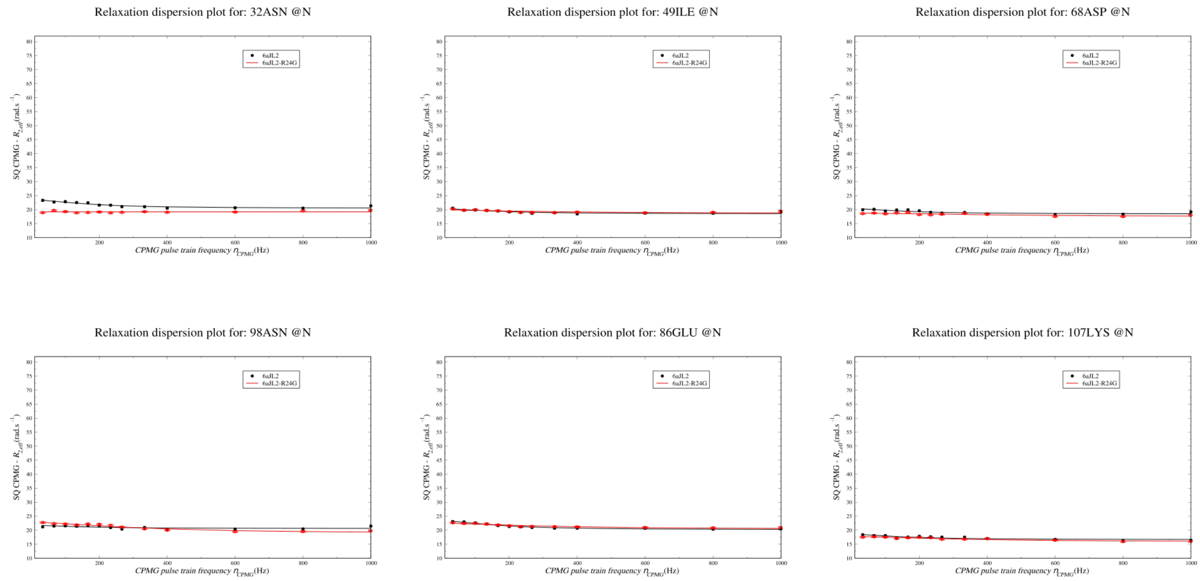

Figure S4. Example of some residues that do not presented exchange for 6aJL2 (black) and 6aJL2-R24G proteins (red). Experiments were performed at 800 MHz 1H frequency.

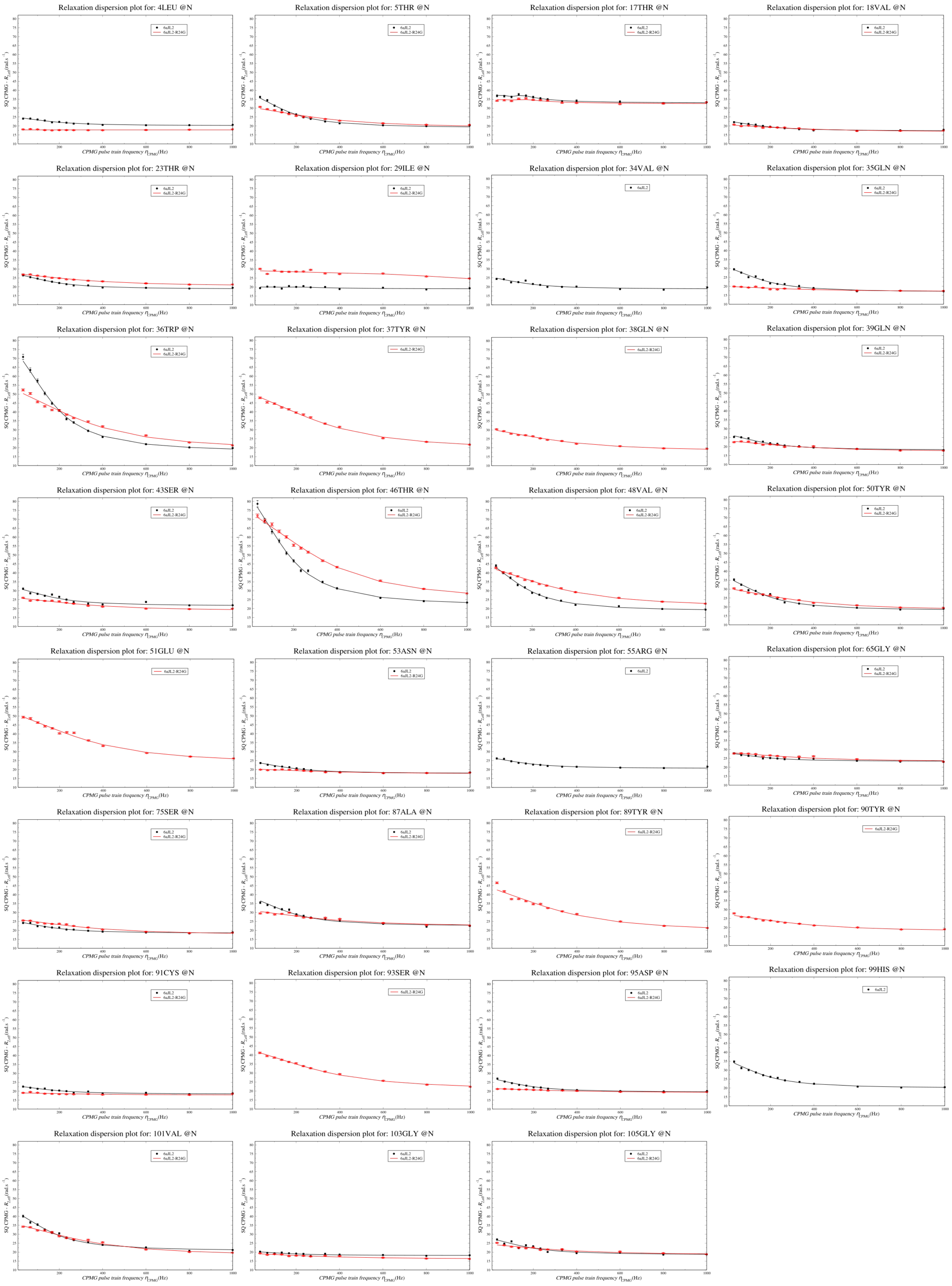

Figure S5. ^{15}N CPMG relaxation dispersion trajectories for 6aJL2 (black) and 6aJL2-R24G proteins (red). Experiments were performed at 800 MHz 1H frequency. Solid line are the fits of CPMG trajectories to a decay exponential function.

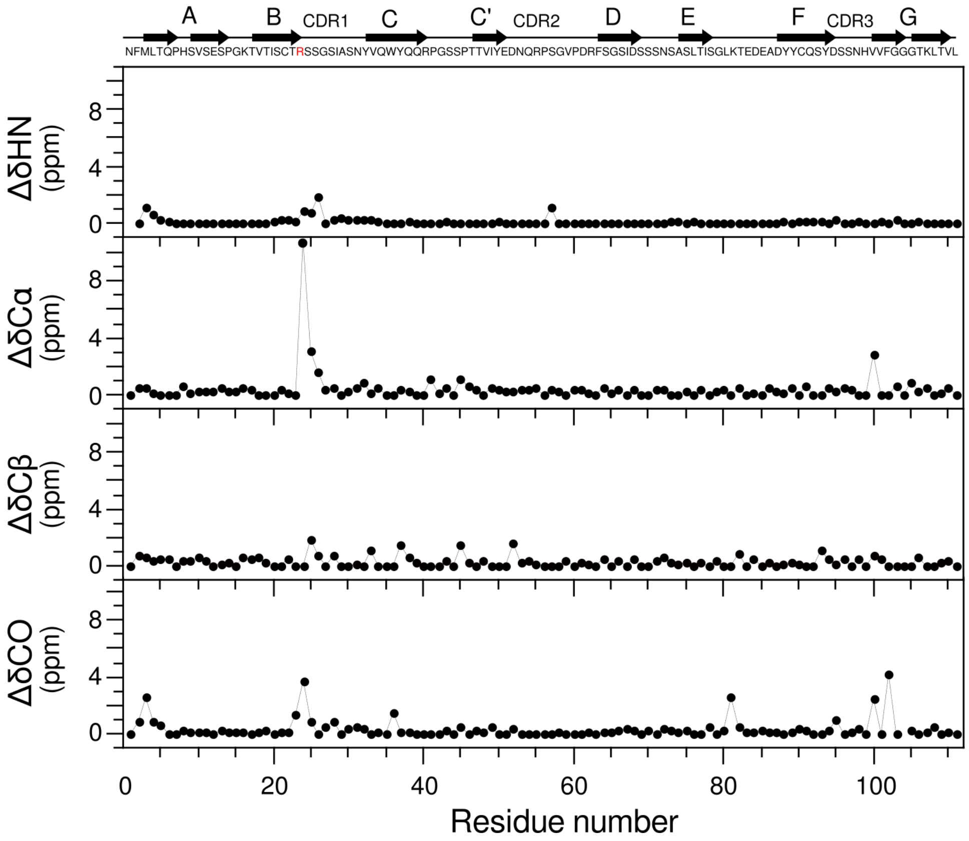

Figure S6. Chemical shifts difference between 6aJL2 and 6aJL2-R24G proteins. Plots of HN, Cα, Cβ and CO differences by residues, respectively from upper to down.

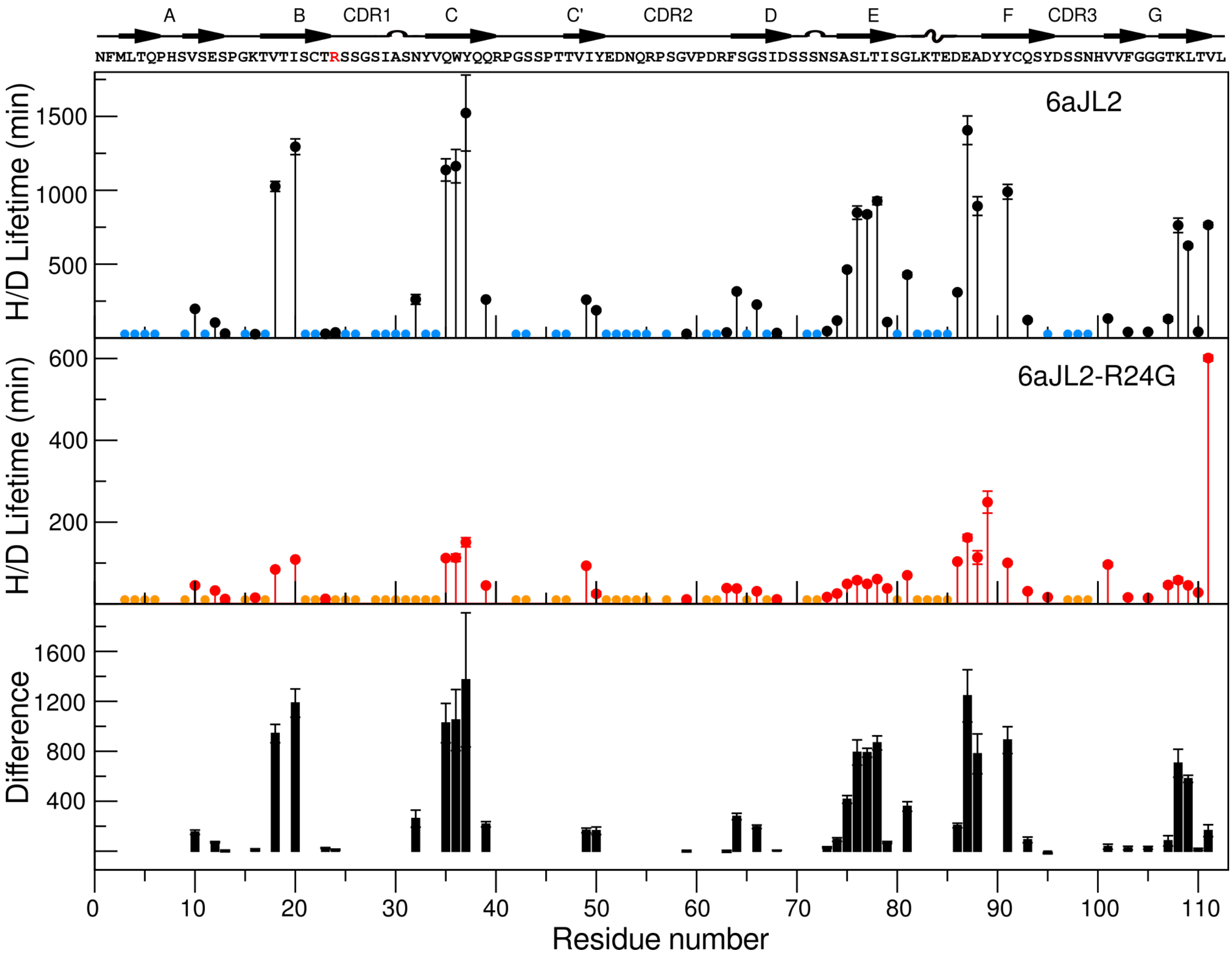

Figure S7. Slow timescale dynamics of 6aJL2 (black) and 6aJL2-R24G (red) followed by hydrogen-deuterium exchange by residues. Rate exchange constant (Rex) were obtained by fitting the intensity decay for each peak. Blue and orange circle represent the residues with higher Rex in 6aJL2 and 6aJL2-R24G proteins, respectively. Black bars are the difference between the Rex of both proteins.
